# Marine-Derived Surface Active Agents: Health-Promoting Properties and Blue Biotechnology-Based Applications

**DOI:** 10.3390/biom10060885

**Published:** 2020-06-09

**Authors:** Ioannis Anestopoulos, Despina-Evgenia Kiousi, Ariel Klavaris, Monica Maijo, Annabel Serpico, Alba Suarez, Guiomar Sanchez, Karina Salek, Stylliani A. Chasapi, Aikaterini A. Zompra, Alex Galanis, Georgios A. Spyroulias, Lourdes Gombau, Stephen R. Euston, Aglaia Pappa, Mihalis I. Panayiotidis

**Affiliations:** 1 Department of Molecular Biology & Genetics, Democritus University of Thrace, 68100 Alexandroupolis, Greece; i.anestopoulos@yahoo.com (I.A.); despina_kiousi@outlook.com (D.-E.K.); arielklvrs@hotmail.com (A.K.); agalanis@mbg.duth.gr (A.G.); 2 Division of Health & Biomedicine, LEITAT Technological Centre, 08005 Barcelona, Spain; mmaijo@leitat.org (M.M.); aserpico@leitat.org (A.S.); asuarez@leitat.org (A.S.); gsanchez@leitat.org (G.S.); lgombau@leitat.org (L.G.); 3 Institute of Mechanical, Process & Energy Engineering, Heriot Watt University, Edinburgh EH14 4AS, UK; k.salek@hw.ac.uk (K.S.); s.r.euston@hw.ac.uk (S.R.E.); 4 Department of Pharmacy, University of Patras, 26504 Patra, Greece; stella.chimic@gmail.com (S.A.C.); azompra@upatras.gr (A.A.Z.); G.A.Spyroulias@upatras.gr (G.A.S.); 5 Department of Applied Sciences, Northumbria University, Newcastle Upon Tyne NE1 8ST, UK; 6 Department of Electron Microscopy & Molecular Pathology, The Cyprus Institute of Neurology & Genetics, 2371 Nicosia, Cyprus; 7 The Cyprus School of Molecular Medicine, PO Box 23462, 1683 Nicosia, Cyprus

**Keywords:** surface active agents, biosurfactants, anti-microbial, anti-oxidant, anti-viral, anti-inflammatory, anti-cancer, anti-aging, blue biotechnology

## Abstract

Surface active agents are characterized for their capacity to adsorb to fluid and solid-water interfaces. They can be classified as surfactants and emulsifiers based on their molecular weight (MW) and properties. Over the years, the chemical surfactant industry has been rapidly increasing to meet consumer demands. Consequently, such a boost has led to the search for more sustainable and biodegradable alternatives, as chemical surfactants are non-biodegradable, thus causing an adverse effect on the environment. To these ends, many microbial and/or marine-derived molecules have been shown to possess various biological properties that could allow manufacturers to make additional health-promoting claims for their products. Our aim, in this review article, is to provide up to date information of critical health-promoting properties of these molecules and their use in blue-based biotechnology (i.e., biotechnology using aquatic organisms) with a focus on food, cosmetic and pharmaceutical/biomedical applications.

## 1. Introduction

Surface active agents (SAAs) are so named due to their capacity to adsorb to oil-water, air-water and solid-water interfaces. SAAs can be further grouped based on their molecular weight (MW) and properties: (i) surfactants are low MW amphiphilic molecules with the ability to lower the surface/interfacial tension between two fluid phases, while (ii) emulsifiers are polymeric molecules that confer longer term stability on the dispersed phase droplets in emulsions and foams [1].

The global SAAs market is steadily growing at a rate that is expected to reach USD 3.21 billion by 2025, with an average annual growth rate (CAGR) of 6.75% [2]. The boost in the application of surfactants has risen along with an increased worldwide environmental awareness, which has translated into a search for more sustainable and biodegradable alternatives, as chemical surfactants are non-biodegradable, and can cause adverse effects on the environment [3,4]. In the last few years, there has been an increased interest in microbial and/or marine-derived molecules, which includes microbially produced and biological SAAs (e.g., biosurfactants) [1]. They are grouped as glycolipids, lipopeptides, fatty acids, polymeric and particulate compounds, of which glycolipids and lipopeptides are the two primary isolated families [5,6]. The metabolic function of biosurfactants in bacteria is that they allow the organisms to use substrates that are not soluble in water, by acting as natural emulsifiers. Biosurfactant-producing bacteria have been widely identified with marine bacteria associated with oil spills and seeps, where they facilitate the uptake of hydrocarbon oils as metabolic carbon sources. Biosurfactants offer several advantages over their chemical counterparts, including lower toxicity, biodegradability, increased environmental compatibility, and good stability at a range of pH and temperatures. Moreover, biological SAAs show associated biological properties that could allow manufacturers to make additional health-promoting claims for their products. Thus, natural SAAs can be multifunctional ingredients with a range of desirable physicochemical and biological properties conferring high added value to consumer products when used in commercial applications. However, their application is hampered by their reduced cost-effectiveness, compared with synthetic SAAs, due to low yields and high production/downstream costs resulting in an average total production cost of about three to ten times higher than chemical SAAs [7]. Recent improvements in the technology of the production of biosurfactants have enabled a 10- to 20-fold increment in productivity. Nevertheless, further improvements are still critical and required [8,9].

The experimental techniques used for the analysis of biosurfactants are varied ranging from thin layer chromatography (TLC) and colorimetric assays to more advanced, high-resolution mass and nuclear magnetic resonance (NMR) spectroscopic analytical methods. Thus, the chemical and structural characterization of lipopeptides, glycolipids and high MW surfactants can be achieved by using a range of analytical procedures based on typical methods for characterization of peptides and fatty acids. Moreover, there are other chromatography techniques used to provide information on characterization and quantification including thin layer chromatography (TLC), preparative TLC (PTLC), column chromatography (CC) or modern semi-preparative/preparative high-performance liquid chromatography (HPLC). Consequently, selection of the appropriate technique(s) should be performed based on the different properties of the target compound including solubility, charge, stability and molecular size. Taking all such information together, the choice of chromatographic methods (and stationary phases to be used) are important for the analysis and/or purification of the marine extract, which could be a complex mixture of lipopeptides, glycolipids, or other biosurfactants. Examples of analytical approaches for the evaluation, identification and structural characterization of surfactants are summarized in Table 1.

The search for marine-derived SAAs has been given impetus by the concept of the “blue economy” which, in turn, encourages a holistic approach to the exploitation and protection of marine environments. In general, our oceans are an immense source and a reservoir of rich and untapped biological resources, but at the same time they have to be exploited responsibly and sustainably. In this review, both biological and functional properties of marine-derived SAAs will be discussed with an emphasis on biological activities which promote health and consequently can form the basis of their use as “key ingredients” in blue-based biotechnology applications.

## 2. Health-Promoting Properties of Marine-Derived SAAs

### 2.1. Anti-Microbial Activity

Marine surfactants have been shown to exert bacteriostatic and bactericidal activities, by destabilizing the cell wall or outer membranes of the pathogens. More specifically, lipidic moieties can be inserted in these structures, changing their charge and morphology, ultimately leading to pore formation and cell death [10]. Furthermore, these interactions with the cellular surface, prevent planktonic cells from forming biofilms, by disrupting quorum sensing mechanisms and preventing attachment to biotic or abiotic surfaces. Lastly, some glycolipid marine-derived biosurfactants can also dislocate and terminate bacterial cells from mature biofilms [11].

There is a plethora of marine-derived lipopeptidic surfactants with antimicrobial potential. The most powerful anti-microbial surfactant, described so far, is surfactin produced by *Bacillus velezensis* H3. To this end, nC_14_- and anteiso-C_15_-surfactins were effective against *S. aureus* and *K. pneumoniae* however, without being able to outperform the antibiotic polymyxin B. On the other hand, their antifungal properties against *C. albicans* were greater than that of vancomycin [12]. In addition, C_15_-surfactin is also synthesized by *Bacillus amyloliquefaciens* strain MB199 and has been shown to act synergistically with ketoconazole against fungal pathogens like *C. albicans*. Nonetheless, as with all surfactins, this isoform also exhibits strong hemolytic activity due to its ability to disrupt membranes [13]. Two other surfactins (CS30-1 and -2) synthesized by *Bacillus* sp. CS30 are also associated with anti-fungal activity against the plant fungus *Magnaporthe grisea*, which causes rice crops to wither, thus resulting in economic loss [14]. Apart from surfactin, other lipopeptides produced by marine microorganisms have also presented anti-microbial and anti-biofilm potential, including lipoptides produced by (i) the marine actinobacterium *Brevibacterium aureum* MSA13 capable of exerting a broad-spectrum antibiotic activity with a most profound effect against *C. albicans* [15]; (ii) a strain of *B. circulans* shown to be more effective than penicillin and streptomycin, especially against *Micrococcus flavus* NCIM 2376, *E. coli* NCIM 2931, *Mycoplasma segmatis* NCIM 5138, *Bacillus pumilus* MTCC 2296, and *Klebsiella* sp. [16], in addition to limiting the growth of other pathogens like *Proteus vulgaris*, *S. aureus*, *A. niger*, and *C. albicans* [17], while a *B. circulans*-producing lipopeptide of the fengycin family had bactericidal effects against *Citrobacter fruendii*, *Alcaligenes faecalis*, *Serratia marcesens*, and *Klebsiella aerogenus* [18]; (iii) the actinobacterium *Nesterenkonia* sp. MSA31, which exhibited anti-biofilm activity against *S. aureus* [19] and *Vibrio harveyi* (a fish pathogen) [20]. Furthermore, aneurinifactin is another novel lipopeptidic surfactant produced by *Aneurinibacillus aneurinilyticus* strain SBP-11 with anti-microbial activity against *K. pneumoniae*, *E. coli*, *S. aureus*, *P. aeruginosa*, *B. subtilis* and *Vibrio cholerae.* This surfactant molecule can anchor to the bacterial membrane, disrupting its continuity and metabolic activity, resulting in the production of hydroxyl radicals, which in turn cause lipid peroxidation and pore formation on the membrane [21]. In addition, the same group isolated and characterized pontifactin, a surfactant lipopeptide produced by a newly identified bacterium, *Pontibacter korlensis* strain SBK-47, that exhibited both antibacterial activity against *Streptococcus mutans*, *M. luteus*, *Salmonella typhi* and *Klebsiella oxytoca*, as well as anti-adhesion potential against *B. subtilis*, *S. aureus*, *S. typhi* and *V. cholerae* [22]. Finally, an amphiphilic lipopeptide (R_n_-Glu^1^-Leu/Ile^2^-Leu^3^-Val^4^-Asp^5^-Leu^6^-Leu/Ile^7^) produced from *Bacillus licheniformis* was capable of demonstrating significant bacteriostatic activity against *E. coli*, *V. cholerae*, *Vibrio paraheamolyticus*, *V. harveyi* but weaker activity against *P. aeruginosa*, *S. aureus* and *Proteus* sp. [23].

The literature also reports the antimicrobial efficacy of glycolipid surfactants produced by marine organisms. To this end, a biosurfactant glycolipid produced by *Streptomyces* sp. MAB36 showed anti-bacterial action against *Bacillus megaterium*, *B. cereus*, *S. aureus*, *E. faecalis*, *Shigella dysenteriae*, *Shigella boydii*, *C. albicans* and *A. niger* [24]. Another glycolipid purified from extracts of the marine halotolerant bacterium *Buttiauxella* sp. M44 also showed anti- *C. albicans*, *A. niger* and anti-*E. coli* activity, as well as mild antagonistic activity against *Salmonella enterica*, *B. cereus*, *B. subtilis* and *S. aureus* [25]. Similarly, another glycolipid produced from the epizootic *Serratia marcescens* (isolated from the hard coral *Symphylia* sp.) exhibited anti-adhesive and inhibitory activity against *C. albicans* BH, *P. aeruginosa* PAO1 and *B. pumilus* TiO1, as well as a damaging potential against pre-formed *B. pumilus* biofilms [26]. On another note, staphylosan, a glycolipid biosurfactant produced by *Staphylococcus saprophyticus* SBPS-15, was documented to possess significant surface tension-lowering activity and complete inhibition of biofilm formation for *B. subtilis* BHKG-7 and *Serratia liquefaciens* BHKH-23. Additionally, it managed to dislodge bacteria from pre-formed *P. aeruginosa* BHKH-19 and *S. liquefaciens* BHKH-23 single-strain biofilms [27]. Lastly, BS-SLSZ2 (a biosurfactant glycolipid isolated from the marine epizootic bacterium *Staphylococcus lentum*) prevented the adhesion of *V. harveyi* and *P. aeruginosa* (two common agricultural pathogens that infect *Altemia salina*), thereby limiting their ability to form biofilms, but without exerting biocidal and/or bacteriostatic activities [28].

Other broad categories of bacterial metabolites with surface-active potential and antimicrobial actions are glycoproteins, peptides and fatty acids. A sponge-associated marine fungus isolated from *Aspergillus ustus* (MSF3) was capable of producing a bioactive glycolipoprotein surfactant with broad-spectrum bacteriostatic activity. More specifically, the ethyl acetate extract of MSF3 was effective in limiting the growth of *C. albicans*, *E. coli*, *M. luteus* and *S. epidermidis* [15]. Additionally, in another study, the marine isolate *B. pumilus* SF214 produced a small molecule of 3 kDa with anti-*S. aureus* activity and surface-active properties which was later identified as pumilacidin composed of a cyclic heptapeptide, a fatty acid with a variable chain length and a peptide (Glu-Leu-Leu-Leu-Asp-Leu-[(Leu/Ile)/Val]) [29]. Similarly, the marine bacteria *Cobetia* sp. MM1IDA2H-1 can be stimulated to produce fatty acids that form micelles, which have been shown to interfere with the fish pathogen *Aeromonas salmonicida* subsp. *Salmonicida*, either by directly affecting the pathogen or by indirectly neutralizing the pathogenic effect via their lipophilic *N*-acyl homoserine lactones. Either way, this does not affect the viability of the pathogens themselves but rather affects biofilm formation [30].

Lastly, some marine microorganisms excrete mixtures of bioactive compounds that have a surface-lowering ability. For instance, the biosurfactant fraction produced by the marine actinomycete strains of *Strepromyces* B3 is a complex mixture of proteins, carbohydrates and lipids possessing anti-microbial activity against *B. subtilis*, *E. coli*, *S. aureus*, *P. aeruginosa* and *C. albicans* [31]. Moreover, *B. amyloliquefacients* SR1 produces a surfactant mixture of surfactin, iturin and fengycin molecules, each of which is known for their surface-active properties and their ability to stabilize emulsions. This mixture was found to be effective in inhibiting phytopathogens like *Rhizoctonia solani*, *Sclerotium rolfsii*, *Fusarium oxysporum* and *Alternaria solani* [32]. In the same way, *Oceanobacillus iheyensis* BK6 (isolated from a naturally occurring biofilm on the coastal region of Sikka, India) produces large amounts of exopolysaccharides (EPS) comprised of mannose, glucose and arabinose. This polysaccharide mixture has emulsifying properties and was shown to inhibit *S. aureus* biofilm formation by disrupting bacteria–bacteria and bacteria–surface interactions, thereby destabilizing the structure of the biofilm [33].

### 2.2. Anti-Oxidantl Activity

An important category of biosurfactants include polysaccharides of marine origin and especially from algae [34,35]. Marine macroalgae are generally classified in three main categories including *Phaeophyceae*, *Chlorophyta* and *Rhodophyta* [36,37]. Among their various constituents (e.g., proteins, lipids, vitamins, minerals and fibers), carbohydrates appear as the most abundant ones including mono-, di-, oligo- and poly saccharides with the latter being further distinguished in sulfated and non-sulfated forms [38]. Over the last few years, many efforts have been directed towards the isolation and identification of new microbial polysaccharides with a variety of functions, including viscosifiers, gelling agents, emulsifiers, stabilizers, and texture enhancers [39].

Recent reports have highlighted the significant antioxidant/free radical scavenging ability of marine polysaccharides with potential beneficial effects to living organisms and the food industry [35,40]. To this end, sulfated (e.g., SCP, ULLP, DAP) and non-sulfated (e.g., GLP) polysaccharide extracts from *Sarcodia ceylonensis*, *Ulva lactuca L*., *Durvillaea antarctica* and *Gracilaria lemaneiformis*, respectively, exhibited significant antioxidant potential and hydroxyl radical scavenging activities [41]. In another study, water-soluble extracellular polysaccharides (e.g., ETW1 and ETW2) have shown significant antioxidant and hydroxyl radical scavenging activities, indicating their potential use as promising antioxidant sources and possible food supplements or ingredients in the field of the pharmaceutical industry [42]. Similarly, the exopolysaccharide EPS273 (isolated from marine bacterium *P. stutzeri* 273) inhibited the formation of biofilms from *P. aeruginosa* PAO1 (an effect that was mediated partially through the reduction of H_2_O_2_ and extracellular DNA production) in addition to preventing the production of hydroxyl and superoxide anion radicals, thereby indicating its strong antioxidant potential [43].

Among different classes of marine-derived polysaccharides, carrageenan is probably the most studied one [44]. This is a hydrophilic sulfated galactan composed of α (1–4)-3, 6-anhydro-D-galactose and β (1–3)-D-galactose units [45] and is considered to be a high-MW polysaccharide with an ester-sulfate content ranging from 15% to 40% [46]. Based on the presence of 3,6-anhydro-bridges, degree of sulfation, extent of solubility, and gelling properties, carrageenans are classified in three main categories: ι (iota), κ (kappa) and λ (lamda) carrageenans, all of which been commercially exploited [47,48]. Their physicochemical characteristics (e.g., emulsifying, stabilizing, gelling and thickening) along with their anti-cancer, [49,50] anti-viral [51], anti-coagulant [52] and anti-bacterial [53] properties enable their use in different industrial sectors (e.g., food, pharmaceutical, cosmeceutical, etc.) [54]. A recent study assessed the antioxidative effects of various isoforms of carrageenan in UVB-induced damage to human keratinocytes (HaCaT) cells, whereby different isoforms of purified carrageenan (e.g., iota 2 (ι2) and iota 5 (ι5) from *Eucheuma spinosum* as well as lambda (λ) and kappa (κ) from *Eucheuma cottonii*) protected against UVB-induced cell death. Moreover, this effect was mediated by the inhibition of free radical formation with a degree of potency following κ > ι > λ suggesting a potential application of carrageenan as a photoprotective skin agent [47]. Furthermore, another study indicated that both native (from *K. alvarezii*) and commercial κ-carrageenans are capable of ameliorating oxidative stress; however, the native type possessed higher antioxidant potential in comparison to the commercial one [55].

Fucoidan is another type of sulfated polysaccharide extracted from marine brown algae consisting of L-fucose and sulfate ester groups [56]. It exhibits a variety of biological activities including anti-inflammatory, anti-cancer, anti-viral and anti-coagulant [57,58]. Interestingly, different extracts of crude fucoidan isolated from brown seaweed *Saccharina japonica* [59] and brown marine alga *Sargassum cinereum* [60] showed important antioxidant activities besides their well-characterized emulsifying and stabilizing capacities. In addition, fucoidan isolated from brown marine alga *Sargassum polycystum* possessed oxidative and nitric oxide radical scavenging activity, reducing power, hydrogen peroxide scavenging activity and total antioxidant capacity [61,62]. Interestingly, in another report, fucoidan ameliorated high-fat diet (HFD)-induced non-alcoholic fatty liver disease (NAFLD) in rats, an activity attributed to the suppression of malondialdehyde (MDA) and nitric oxide (NO) levels as well as an increase in total glutathione (GSH) levels, accompanied by inhibition of important inflammatory mediators like interleukin (IL)-1β, and tumor necrosis factor (TNF)-α [63]. Moreover, in a study where 33 morphologically distinct actinomycetes were isolated from the marine sponge *F. cavernosa* and screened for biosurfactant production, the actinobacterial strain *Nesterenkonia* sp. MSA31 (isolated from the marine sponge) exhibited high antioxidant potential [19].

Another marine-derived cyclic lipopeptide surfactin was demonstrated to possess antioxidant activity by inhibiting amyloid-β-induced ROS production and reducing the production of important inflammatory components like TNF-α, IL-1β, IL-6, monocyte chemoattractant protein (MCP)-1 and prostaglandin E2 (PGE2), in BV2 microglia cells, suggesting that this lipopeptide could be used in the context of a therapeutic means against neurodegenerative disorders involving neuroinflammation as part of their pathophysiology [64,65]. Finally, the isolated marine *Streptomyces* sp. N11 strain was found to produce a biosurfactant with significant total antioxidant capacity [66].

### 2.3. Anti-Viral Activity

Naturally derived anti-virals are divided in two major classes: (i) directly acting and (ii) host-acting antivirals (DAAs, HAAs respectively) [67]. On the other hand, surface-active agents are interesting candidates for anti-viral drug discovery, either as DAAs or HAAs, due to their physicochemical properties. The most profound viricidal effect of surface-active agents is the destruction of the lipid membrane of enveloped viruses. Consequently, viral infectivity is diminished as their early infection stages of attachment and internalization are impaired. Interestingly, SAAs can also interfere with the later stages of the viral life cycle, namely: (i) gene expression, (ii) virion assembly, and (iii) exit. Lastly, surfactants targeting host gene expression and signaling cascades could limit viral activity, indirectly, by enhancing the host’s response and improving viral clearance.

Various reports have indicated the activity of marine-derived biosurfactants against viral attachment and entry. For example, carrageenans have been shown to inhibit the attachment of different strains of herpes simplex virus (HSV) [68,69], influenza virus H1N1 [70], human papilloma virus (HPV) [71] and enterovirus-71 [72]. For instance, in the case of HPV, sulfated polysaccharides mimic the structure of heparin sulfate (the receptor that HPV contacts before its internalization in the host cell) thereby acting as decoy receptors [73,74].

On the other hand, co-incubation of fucoidans, isolated from 3 marine brown seaweeds (e.g., *Sargassum mcclurei*, *Sargassum polycystum* and *Turbinara ornate*), and a sulfated fucan derived from the brown seaweed *Cystoseira indica* have all shown potency in inhibiting the attachment of human immunodeficiency virus (HIV) and HSV viruses [75,76]. In another study, fucoidan isolated from the marine alga *Cladosiphon okamuranus* directly interacted with the envelope glycoprotein of dengue virus serotype 2 (DENV-2), impairing its attachment and internalization to the host cell [77]. Furthermore, given that enveloped viruses can also be inactivated after disruption of their lipid membranes, it was shown that sophorolipids were potent in limiting HIV infections [78].

Similarly, surfactin was shown to disrupt the lipid membranes of various enveloped viruses and partially disrupt their capsid [79]. In addition, surfactin was also proven to act as a fusion inhibitor against porcine epidemic diarrhea virus (PEDV) and transmissible gastroenteritis virus (TEDV) [80]. Moreover, other studies have shown that carrageenans and fucoidan inhibited the internalization and uncoating of DENV-2, -3, rhinovirus (HRV) [81], DENV-2 and Newcastle disease virus (NDV) [82,83]. Lastly, fucoidan was shown to inhibit cell-to-cell transmission of Human T-lymphotropic virus type-1 (HTLV-1), in cell cultures, suggesting a promising therapeutic and/or prevention approach [84].

Finally, another antiviral mechanism of action by marine SAAs is mediated through alteration of host cell gene expression. Interestingly, iota-carrageenan showed differential antiviral effects against DENV, which appeared to be specific to the cell culture model used in each case. This polymer postponed viral attachment on eukaryotic cells; however, in mosquito cell cultures, iota-carrageenan exerted its viricidal effects by altering host gene expression. The antiviral effects exerted in the mosquito cells are believed to stem from the carrageenan-induced alterations in host factors that play a role in successful DENV infections; however further studies are necessary [85]. Lastly, in another study, it was shown that kappa carrageenan halted the enterovirus-71-induced apoptotic death in infected Vero cells suggesting an anti-apoptotic effect, which could be further explored as a promising therapeutic approach for the alleviation of various disease manifestations [72].

### 2.4. Anti-Inflammatory Activity

The literature has attributed many biological functions to surfactin, including an anti-inflammatory potential. To this end, a recent study reported a surfactin-mediated reduction of pro-inflammatory mediators in lipopolysaccharide (LPS)-induced macrophage stimulation. Specifically, surfactin inhibited the activation of NF-κB by blocking the degradation of its cytoplasmic suppressor namely the inhibitor of nuclear factor kappa-B kinase subunit beta (IKK-β), an effect accompanied by a dose-dependent reduction of the transcriptional levels of interferon (IFN)-γ, IL-6 and inducible nitric oxide synthase (iNOS) [86]. In addition, surfactin-induced inhibition of NF-κΒ, PI3K/Akt, p38 kinase and c-Jun N-terminal kinase (JNK), resulted in down-regulation of (i) major histocomplex-II (MHC-II) receptors, (ii) costimulatory molecules, and (iii) expression of interleukin IL-12p70 leading to significant limitation of macrophage ability to present antigens to and activate CD4+ T cell populations [87]. Consistent with these results, surfactin also exerted neuroprotective and anti-inflammatory effects on lipoteichoic acid-stimulated BV-2 microglial cells. More specifically, it activated the cyclic adenosine 3’, 5’-monophosphate (cAMP)-protein kinase A (PKA)-cAMP response element-binding protein (CREB) pathway and suppressed the activation of NF-κΒ. As a result, the subsequent activation of heme oxygenase (HO-1) and nuclear factor-like-2 (Nrf-2) orchestrated the anti-inflammatory and anti-oxidant effects that were reflected as lower levels of the inflammatory markers TNF-α, IL-1β, IL-6, MCP-1, prostaglandin E2 (PGE2) and nitric oxide (NO), as well as reactive oxygen species (ROS) [65].

Marine polysaccharides have also been reported for their anti-inflammatory properties. A sulfated polysaccharide (derived from the brown seaweed *Sargassum horneri* and with a structure similar to commercial fucoidan) downregulated the expression of iNOS and cyclooxygenase-2 (COX-2), as well as TNF-α and IL-1β, by inhibiting p38, mitogen-activated protein kinase (MAPK) and NF-κΒ in LPS-stimulated RAW 264.7 macrophages [88]. In another study, the in vitro anti-inflammatory activity of fucoidan (purified from *Fucus vesiculosus*) was documented against LPS-induced inflammation in BV2 microglial cell line. Specifically, fucoidan treatment reduced the production of NO, PGE_2_ and the pro-inflammatory cytokines IL-1β and TNF-α, by down-regulating iNOS, COX-2, MCP-1, NF-κB expression levels and disrupting the MAPK and AKT signaling pathways [89]. In fact, several in vivo studies have also confirmed the ability of different sulfated polysaccharides to exert anti-inflammatory effects. To this end, fucoidan (isolated from the marine brown algae *Turbinaria ornate*) exhibited an anti-inflammatory action against cotton-pellet induced granulomas in rats, comparable to dexamethasone (an established anti-inflammatory agent). Following fucoidan treatment, inflammatory markers including cathepsin D, myeloperoxidase (MPO) and C-reactive protein (CRP) showed trends of a declined expression pattern [90]. Fucoidan-mediated reduction of the above-mentioned inflammatory markers resulted in delayed recruitment of leukocytes to the inflamed region, after halting their extravasation process [91,92]. Lastly, fucoidan treatment reversed inflammation-related tissue damage in an ex vivo human skin experimental model, by down-regulating the expression of elastase, a leukocyte enzyme responsible for the proteolytic degradation of connective tissue during inflammation [93].

**Figure 1:**
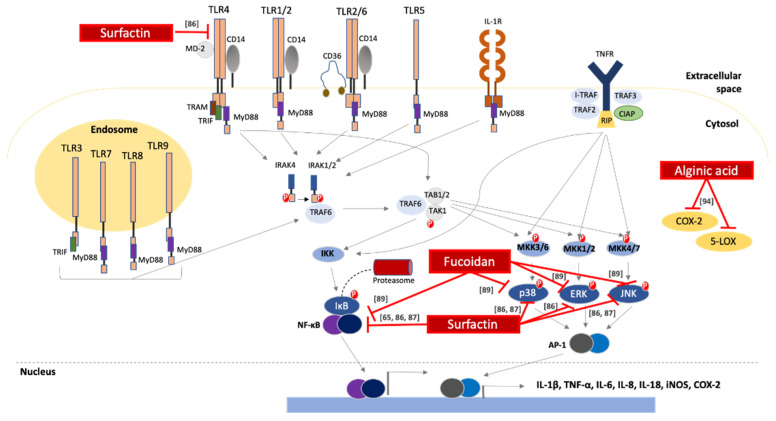
Anti-inflammatory activity of marine-derived SAAs. Biosurfactants halt the production of pro-inflammatory mediators by interfering with pathways induced by the stimulation of Toll-like receptors, interleukin-1 receptor and Tumor Necrosis Factor-α receptor. These compounds directly inhibit key molecules of the IKK/NF-κΒ, p38 MAPK, MAPK/ERK and JNK signaling pathways or inactivate cyclooxynase-2 (COX-2) and 5-lipoxygenase (5-LOX) that catalyze the production of inflammatory molecules. TLR, Toll-like receptor; MD-2 (encoded by the *LY96* gene): lymphocyte antigen 96; IL, interleukin; IL-1R, interleukin-1 receptor; MyD88, myeloid differentiation primary response 88; CIAP, calf intestinal alkaline phosphatase; RIP, ribosome-inactivating protein; TRIF, toll/interleukin-1 receptor-like protein (TIR)-domain-containing adaptor-inducing interferon-β; ΤRAM, TRIF-related adaptor molecule; TRAF, tumor necrosis factor receptor-associated factor; TAB1/2, tumor growth factor-β (TGF-β)-activated kinase 1; TAK, TGF-β activated kinase 1; IKK, IκΒ kinase; MKK, mitogen activated protein (MAP) kinase; COX-2, cyclooxygenase-2; 5-LOX, 5-lipoxygenase; iNOS, inducible nitrogen oxide synthase; NF-κΒ, nuclear factor κΒ; ΕRK, extracellular signal activated kinase; JNK, c-Jun N-terminal kinase; AP-1, activator protein-1; IRAK, IL-1 receptor associated kinase.

Finally, alginic acid (a non-sulfated polysaccharide of marine origin) has been long known for its immunomodulatory and emulsifying properties. In fact, a recent study has described the anti-inflammatory properties of alginic acid (isolated from the marine algae *Sargasum wightii*) including reduction of the symptoms of Collagen type II-induced arthritis, in rats, by lowering the levels of prostaglandins and leukotrienes [94]. Of note, alginic acid was more potent in reducing signs of inflammation compared to indomethacin (a non-steroidal drug prescribed to treat inflammation in various pathological conditions) [94]. The underlying molecular mechanisms mediating the anti-inflammatory activity of marine-derived biosurfactants are summarized in Figure 1.

### 2.5. Anti-Cancer Activity

Various SAAs have been described to exert anti-cancer properties [95]. To this end, surfactin was shown to exert an anti-cancer potency against colon cancer (LoVo) cells by inhibiting their proliferation through mediation of pro-apoptotic activities and cell cycle arrest via suppression of extracellular signal-regulated kinases (ERK) and phosphatidyl inositol-3 kinase (PI3K)/v-akt murine thymoma viral oncogene homologue: protein kinase B (AKT) cell-survival signaling pathways [96]. A purified biosurfactant product (containing surfactin and fengycin isoforms; isolated from *Bacillus circulans* DMS-2 (MTCC 8281)), was shown to possess selective anti-proliferative activity against human colon tumor (HT-29 and HCT-15) cell lines [97]. Likewise, two fengycin isoforms, named iso-C16 fengycin B and anteiso-C17 fengycin B, extracted from marine *Bacillus mojavensis* B0621A, induced cytotoxicity against human leukemia (HL-60) cells [98].

Iturin A, isolated from marine bacterium *Bacillus* megaterium, was shown to inhibit growth of MDA-MB-231 and MCF-7 breast cancer cells through reduction of phosphorylated Akt kinase levels in addition to exerting an anti-cancer capacity against MDA-MB-231 xenograft model in nude mice. The anti-cancer capacity was documented as tumor volume reduction, decreased expression levels of Ki-67 (a marker of proliferation), cluster of differentiation 31 (CD-31), phospho-Akt, P-MAPK, phosphorylated glycogen synthase kinase-3 beta (P-GSK3β) and phosphorylated fork-head box class O 3α (P-FoxO3α) [99]. Moreover, iturin A extracted from the same bacterium strain, re-sensitized docetaxel resistant MDA-MB-231 and MDA-MB-468 breast cancer cells by reducing phosphorylated-Akt expression levels thus leading to the subsequent inactivation of Akt [100].

Rhamnolipids, a class of glycolipids derived from marine bacteria *Pseudomonas aeruginosa*, were also reported for their anti-tumor capacity [23]. In a recent study, the cytotoxic effect of three different monorhamnolipids (Rha-C10-C10, Rha-C10-C12, and Rha-C14-C10), isolated from the arctic marine *Pseudomonas* sp. strain M10B774, was reported against human melanoma (A2058), breast (MCF-7) and colon (HT-29) cancer cell lines by unidentified underlined mechanisms [101].

Additionally, several marine-extracted polysaccharides have been reported for their anti-cancer properties against various cancer cell lines. Fucose-containing sulfated polysaccharides (isolated from brown algae) have been associated with promising anti-cancer activity via different mechanisms: (i) inhibition of cell growth and proliferation through cell cycle arrest (after induction of the G1 phase via inhibition of phosphorylation of the retinoblastoma (RB) protein and/or up-regulation of the cyclin-dependent kinase (CDK) inhibitors p21WAF1/CIP1 and p27KIP1) [102,103,104]; (ii) apoptotic induction [103] through activation of the intrinsic pathway (via regulation of Bcl-2/Bax expression levels [105] and down-regulation of endogenous inhibitors of caspases-3 and -9, X-linked inhibitor of apoptosis protein (XIAP) and Livin (a novel inhibitor of apoptosis protein family member), respectively [106]; (iii) anti-metastatic ability mediated by the inhibition of the proteolytic activities of matrix metalloproteases (MMPs)-2 and -9 [107].

**Figure 2:**
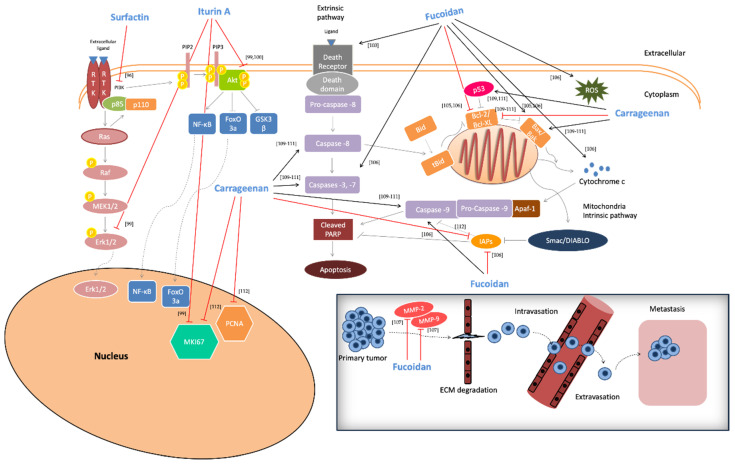
Anti-cancer activity of marine-derived SAAs through induction of both intrinsic and extrinsic apoptotic pathways. Biosurfactants exhibit considerable anti-cancer activity by inhibition of the MAPK/ERK and Akt/PI3K signaling pathways, as well as through suppression of nuclear antigens, MKI67 and PCNA. Their anti-cancer capacity is also mediated through reduced expression of MMPs-2 and -9 known to be associated with tumor metastasis. RTK, Receptor tyrosine kinase; PI3K, Phosphatidylinositol-3-Kinase; MAPK, Mitogen-activated protein kinase; ERK, Extracellular signal–regulated kinase; MEK, MAPK/ERK kinase; PIP_2_, Phosphatidylinositol 4,5-bisphosphate; PIP_3_, Phosphatidylinositol (3,4,5)-trisphosphate; NF-kB, Nuclear factor kappa-light-chain-enhancer of activated B cells; FOXO3a, Forkhead box O3a; GSK3β, Glycogen synthase kinase 3 beta; MKI67, Marker of Proliferation Ki-67; PCNA, Proliferating Cell Nuclear Antigen; PARP, Poly (ADP-ribose) Polymerase; Bid, BH3-interacting domain death agonist; tBid, truncated Bid; Bcl-2, B-cell lymphoma 2; BCL-XL, B-cell lymphoma-extra-large; BAX, BCL2 associated X; BAK, BCL2-antagonist/killer; ROS, Reactive oxygen species; Smac/DIABLO, Second mitochondria-derived activator of caspase/direct inhibitor of apoptosis-binding protein with low pI; IAPs, Inhibitors of apoptosis protein; Apaf-1, Apoptotic protease activating factor 1; MMP-2, Matrix metalloproteinase-2; MMP-9, Matrix metalloproteinase-9; ECM, Extracellular matrix.

**Figure 3:**
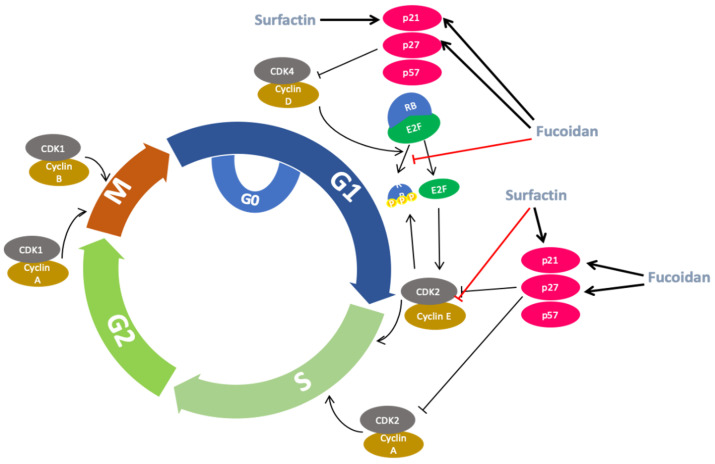
Anti-cancer activity of marine-derived SAAs through regulation of cell cycle progression. Surfactin and fucoidan exhibit anti-cancer activity by blocking cell cycle progression through up-regulation of inhibitor(s) levels of CDKs. In addition, fucoidan induce G1 phase cell cycle arrest, through inhibition of RB phosphorylation. pRB, phosphorylated retinoblastoma; CDKs 4,2,1, Cyclin-dependent kinases 4,2,1; E2F, E2 promoter binding Factor.

Another type of sulfated polysaccharide, carrageenan, has been reported for its anti-cancer capacity by exhibiting significant anti-proliferative ability [against several cancer cell lines including MCF-7 (breast cancer), HT-29 (colon cancer), HepG-2 (liver cancer), MG63 (osteosarcoma cancer) and SH-SY5Y (human neuroblastoma)] via unidentified underlined mechanism(s) [55,108]. Moreover, the anti-tumor activity of ι-, λ- and κ-carrageenans was demonstrated to be mediated through (i) increased levels of Bax: Bcl-2 ratio, (ii) promotion of caspase-dependent apoptosis [109,110,111], and (iii) inactivation of proliferating cell nuclear antigen (PCNA) and marker of proliferation Ki-67 (MKI67) along with the down-regulation of surviving, all of which are involved in the progression of cell proliferation [112]. Finally, carrageenan isolated from different marine red algae strains (e.g., *Kappaphycus alvarezii*, *Chondrus ocellatus* and *Kappaphycus striatum*) demonstrated in vivo anti-cancer capacity against tumor-bearing mice. Specifically, solid-type tumor sarcoma 180 (S180) transplanted in mice appeared reduced in volume following treatment with carrageenan. In parallel, an increase of (i) spleen and thymus volume, (ii) macrophage phagocytosis, (iii) proliferation activities in lymphocytes and natural killer (NK) cells, and ultimately, (iv) higher expressions of TNF-α and IL-2 were recorded, all of which are indicative of induced activation of the immune system [49,113,114]. Finally, a similar action of carrageenan was observed in an in vivo model of melanoma B16-F10 cells injected in C57BL/6 mice [115]. In conclusion, marine-derived surfactants appear to exert considerable anti-cancer activities in both in vitro and in vivo models, targeting a variety of molecular components implicated in major signaling and cell cycle progression pathways, as summarized in Figure 2 and Figure 3.

### 2.6. Anti-Aging Activity

The pleiotropic biological effects of marine-derived SAAs also include their ability to attenuate or even prevent skin-photoaging, suggesting an important role in skin care research. Fucoidan and carrageenan sulfated polysaccharides have been associated with significant beneficial effects against skin disorders [34,116,117]. To this end, fucoidan (isolated from *Costaria costata*) has been shown to possess anti-aging activity against an in vitro model of UV-B induced photo-damage in human immortalized keratinocyte (HaCaT) and foreskin fibroblast (HS68) cells mediated via suppression of (i) mRNA and protein expression of MMP-1 and (ii) ERK and JNK signaling pathways, ultimately resulting in increased levels of type 1 pro-collagen synthesis [118,119,120]. Similarly, a 16 kDa fraction of fucoidan (isolated from the brown algae *Ascophyllum nodosum*) was found to interfere with different factors contributing to the breakdown of connective tissue including (i) inhibition of the production of gelatinase A (MMP-2) and stromelysin-1 (MMP-3) expression induced by IL-1β, and (ii) increased levels of metallopeptidase inhibitor 1 (TIMP-1), together with those reduced by human leukocyte elastase (HLE), collectively resulting in protection of dermal elastic fiber network [93].

Accordingly, the beneficial effects of fucoidan (isolated from *Mekabu* alga) were reported through reduced expression levels of MMP-1 and IFN-γ, in UV-B irradiated mice, accompanied by suppression of edema and reduction of prickle cell layer thickness [121]. Of note, low MW fucoidans (LMF; isolated from *Sargassum hemiphyllum*) prevented UV-B induced damage in human foreskin fibroblast (HS68) cells by inhibiting UVB-induced AP-1 mediated stimulation of collagenases (MMP-1, -8, and -13) and gelatinases (MMP-2 and -9), along with the positive regulation of TGFRβ receptor II mRNA levels, thus preventing collagen degradation [122]. Finally, different isoforms of carrageenans—e.g., ι (II) and ι (V) obtained from *Eucheuma spinosum*, and λ and κ (III) obtained from *Eucheuma cottonii*—have also been reported to exhibit a photoprotective properties by inducing cell death of UV-B-irradiated human keratinocyte (HaCaT) cells, thereby indicating a novel role of carrageenans as anti-photodamage agents [47].

## 3. Blue Biotechnology-Based Applications of Marine-Derived SAAs 

### 3.1. Food Applications

Surfactants and emulsifiers are exploited in foods for their surface active properties (which aid in the formation of emulsions and foams) to control dough strength in bread, modify viscosity and control fat crystallization [123,124,125,126]. Emulsifiers used in foods include proteins, polysaccharides or their varying amalgams of the two (e.g., proteoglycans, glycoproteins, etc.). For polysaccharides, the term emulsifier is something of a misnomer, and most are better described as stabilizers and while most polysaccharides can stabilize emulsions, only a few are able to emulsify oils. This is because the majority lack an amphiphilic structure, are not surface active and cannot adsorb to fluid interfaces. The stabilizing ability of polysaccharides is associated with their ability to thicken solutions, which slows down destabilization of emulsions and foams [127].

The most widely used surfactants in the food industry are mono- (MGs) and diglycerides (DGs), either mixed (MDGs), or as distilled monoglycerides (MAGs) [126,128]. Although MDGs are the most used of food surfactants, they are under scrutiny due to perceptions of their environmental impact. Palm oil is a major source of triglycerides used in the production of MDGs. However, it has received considerable attention in the press due to extensive deforestation activities in South East Asia to enable establishment of palm oil plantations [129,130], and thus MDGs are under scrutiny due to perceptions of their environmental impact. Deforestation has led to extensive loss of habitat for several critically endangered species in the locale, especially the orangutan. Food manufacturers have responded, driven by consumer pressure, through reduced use of palm oil-derived products including MDGs. The concern over the sustainability and environmental impact of palm oil products is such that the European Union (EU) has considered a ban on the use of non-sustainably produced palm oil. Thus, MDGs are a major target for replacement with environmentally friendly, sustainable biosurfactants.

MDGs are the main surfactants used in baked goods (breads, cakes, biscuits). In these products, the functions of surfactants are (i) emulsification of bakery fats (shortening); (ii) synergistic interactions with flour; (iii) enhancement of the properties of the shortenings; and (iv) improved aeration [123,125,126]. MAGs are also found in several dairy products including ice-cream and whipping cream mixtures where surfactants are added to help stabilize the initial foam and to destabilise fat emulsion droplets during aeration [124], to act as nucleation points for the crystallization of triglycerides during cooling and to help in the air-incorporation process by promoting partial coalescence of fat globules both at the air bubble surface and in the bulk of the ice-cream/cream [124].

A range of surfactants have several roles in sugar confectionary, as emulsifiers in toffee, fudge and caramel [131], as a lubricant to control viscosity and flow properties in chocolate processing and to control fat crystallization, and in particular to reduce fat bloom, a quality flaw in chocolate.

There are many examples where biosurfactants have been suggested to be potential emulsifiers, foamers and surface tension lowering agents [132,133,134,135] in food applications, but to date there have been very few reports where this has been explicitly demonstrated in food formulations. The most common food systems where biosurfactants have been trialled are baked goods. This is perhaps not surprising as it has been known for over 60 years [136] that galactolipids found in wheat flour act as surfactants and improve the volume, texture, and reduce staling of bread [137,138]. This suggests that glycolipids could be useful replacers for MAGs that are added to bread for the same function. While the galactolipids in wheat flour are not marine derived, galactolipids are common to all photosynthetic organisms, being found in the thylakoid membrane of the chloroplast [139]. Thus, photosynthetic marine microorganisms such as microalgae and cyanobacteria are potential sources of galactolipids, although to date they have not been widely explored nor exploited for this. Reports of the use of biosurfactants in foods are not explicitly for those derived from marine organisms, although biosurfactants of the types tested in foods have been identified from marine bacteria. Patented applications of rhamnolipids (although not explicitly from a marine source) in bakery products include improvement of dough stability in breads and batter stability in cakes, increased volume of bread loaf or cakes, a better structure of the crumb or crust, and an overall improvement in the shape of the product. The improved dough or batter stability gives the product additional resistance to mechanical shock during the process, so the product is less likely to collapse during fermentation or baking. When compared to common synthetic surfactants such as DATEM (diacetyl tartaric acid ester of mono- and diglycerides), which is used at levels of 0.1–0.5% to impart these improvements, rhamnolipids require only 0.025% addition levels for the same effect [140]. A similar improvement in bread quality was noted when the lipopeptide surfactant SPB1 (from *Bacillus subtilis*) was substituted for lecithin at a concentration of 0.075%. SPB1 also provided a significant anti-staling and antimicrobial effect [141]. In addition, another study has reported an improvement in cookie dough texture and overall baked cookie quality when the same SPB1 lipopeptide is used in place of GMS as a dough improver [142].

Polysaccharides have a range of functions in foods that are closely linked to the way they interact with water. These functions include thickening, gelling and water retention, and some are also used for their emulsifying ability. A common name for polysaccharides is the term stabilizer, indicating that these functionalities lead to improved stability of the food, e.g., reduced separation of ingredients or water [143]. Mono- and diglycerides have not been reported as a major metabolite of marine microorganisms. However, there are several prolific producers of polyunsaturated oils among marine microorganisms, including microalgae and protists (thraustochytrids) [144,145]. This does offer the opportunity to use marine microbial oils as a feedstock for the production of mono- and di-glycerides through controlled glycerolysis [146].

Polysaccharides have been used widely in the baking industry for many years for their ability to control dough rheology, anti-staling and to slow the retrogradation of starch, which leads to a greater product volume, softer texture and longer shelf-life [147,148]. Although many different polysaccharides have been reported as being used in baked goods, there is little difference in the functions that these have, and little reported difference in their overall effect [148]. Polysaccharides derived from marine algae are well represented as food ingredients. Agar, alginates and carageenans are a common ingredient in bakery formulations. Agar is added to baked goods due to its ability to modify the properties of starch, its thermostability [149], and its water holding capacity that increases the moisture in bread and improves crust texture and color [150]. Alginates have a similar effect on the quality of baked goods, as well as providing an additional anti-staling effect [151], which is also closely linked to its enhanced water holding capacity. Carageenans are anionic polysaccharides that require a divalent cation (often calcium) to gel. When added to bread dough, the interactions between the carageenan and gluten proteins have a synergistic effect on bread quality [152].

In dairy products such as chocolate milk, polysaccharides form a weak gel entrapping the cocoa particles and fat emulsion droplets thus preventing their sedimentation or creaming [153]. Thickening properties are also exploited in ice-cream to produce a smooth texture [154], with an added advantage that the increased viscosity helps in the incorporation of air during whipping. The water binding ability of polysaccharides helps to control ice-crystal size during freezing and subsequent temperature cycling and reduces the likelihood of a gritty texture to the product [154].

Although polysaccharides from marine algae are routinely used in foods, marine bacterial polysaccharides, on the other hand, although many have been identified [155], have not been taken beyond lab-scale production, and their use in foods at industrial scale has not as yet been implemented. There are several studies that have reported properties that are valued by food manufacturers, including emulsification, thickening/gelation and anti-freeze properties [155]. The most studied microbial emulsifier from marine bacteria, and the one that has come closest to successful exploitation, is emulsan, produced by *Arthrobacter* sp. RAG-1 [156]. Emulsan is a lipo-heteropolysaccharide containing D-galactosamine, L-galactosamine uronic acid, and a diamino, 2-desoxy n-acetylglucosamine, decorated with ester and amide linked fatty acids [156]. The surface activity and emulsifying properties come from the hydrophobic lipid chains, but this is also enhanced by the presence of 10–20% of proteinaceous material in the crude emulsan extract [157]. The good emulsifying and surface tension lowering ability led to several patents being granted for emulsan applications (see for example patents EP0178443B1 & EP0242296A1/fi). Despite this, there are no reported applications in foods. One potential drawback that may have held back the exploitation of emulsan is the reported rapid reduction in emulsifying ability below pH 4.0 due to loss of charge on the uronic acid groups (pKa 3.05). Many foods are acidic, which is advantageous for microbial safety and stability, and emulsan would not necessarily be a useful emulsifier under these conditions.

A non-exhaustive summary of marine microbial emulsifiers having properties that could be of interest to food manufacturers is given in Table 2. Of the reported functional properties, emulsification is the most common, with many bio-emulsifiers also showing the ability to thicken and gel solutions. Foaming properties are rarely reported. One other common property is cryoprotection. This relates to the ability of polysaccharide bio-emulsifiers to interact with water and would be a property of interest to manufacturers of frozen products such as ice-cream.

### 3.2. Cosmetic Applications

The cosmetic industry is a growing sector believed to reach a value of 430 billion dollars by 2022 [181]. To be able to assume this volume of business, the sector needs new sources of assets that are mainly oriented towards the search for ingredients of natural origin [181]. One of the fundamental components of the cosmetic formulation are surfactants which are used to (i) either eliminate (cleansing) or add (emulsification) oils to the skin or hair; (ii) produce foam; (iii) obtain transparent solutions (solubilization); (iv) improve the appearance and touch after application (conditioning); and as (v) preservatives (assuming they possess anti-microbial properties) [182]. Chemical surfactants are classified according to the type of the polar group present, including: (i) anionics, (ii) acyl-amino acids and salts, (iii) cationic, (iv) amphoteric surfactants, and finally (v) non-ionic surfactants, widely used as cleansing, foaming, emulsifiers, stabilizers and thickening agents [183]. However, most of the surfactants used by the cosmetic industry have been synthesized from petroleum derivatives, entailing biodegradability (due to the action of microorganisms), bioaccumulation and biocompatibility issues for the environment as well as the human health. In the latter case, several studies have demonstrated that synthetic surfactants can alter the natural barrier of the skin (causing damage and irritation) as it is the case for sodium lauryl sulphate (SLS) and sodium laureth sulphate (SLES), two widely used surfactants in the formulation of cosmetic products [184,185,186]. To this end, and based on the growing interest (driven by consumer demand and new environmental control legislation), cosmetic companies have switched to labelled “sulphate-free” shampoos by replacing surface-active chemical agents by naturally derived counterparts (biosurfactants). Indeed, a variety of biosurfactants (e.g., saponins, surfactin as well as rhamnolipid, sophorolipid and mannosylerythritol lipids (MEL)) have been widely used in cosmetic formulations as anti-aging, anti-oxidant and anti-microbial ingredients possessing a safe profile with low toxicity [186,187].

Among the potential characteristics described for biosurfactants, their property as emulsifiers is one of the most relevant for cosmetics since it facilitates the application of the product and can lower its cost given that water is the major component of such solution. For instance, *Nesterenkonia* sp. MS31 is an actinobacterium isolated from the marine sponge *Fasciospongia cavernosa*. This strain produces a lipopeptide with emulsifying capacity that is thermo-stable at a range of temperatures (4.0–121.0 °C), which is of great value in the formulation and preservation of cosmetic products [19]. Furthermore, Liposan and Yansan are biosurfactants synthesized from the Brazilian marine strain of *Yarrowia lipolytica*, IMUFRJ50682, when grown on n-alkanes or aliphatic/aromatic hydrocarbons and perfluorocarbons respectively. Although both molecules form stable oil/water emulsions, Yansan has a higher emulsification activity and stability ranging from very acidic (pH 3.0) to very basic (pH 9.0) [188,189]. Evaluation of how acidic or basic a solution is, represents a very important property for toothpaste formulation since an acidic pH can cause an abrasive effect and deteriorate dentin while a neutral or basic pH is essential for good oral health. Toothpaste formulation with a biosurfactant obtained from the marine actinobacteria *Nocardiopsis* VITSISB showed a more basic pH optimum than that formulated with SLS the most commonly commercially used biosurfactant [190].

In summary, data from numerous studies support the use of biosurfactants for skin care and cosmetic products formulation based on their relevant properties including, but not limited, biodegradability, biocompatibility and various relevant biological activities (e.g., antioxidant, anti-wrinkle, ant-aging, etc.).

### 3.3. Pharmaceutical/Biomedical Applications

In pharmaceutics and medicine, the use of biosurfactants is aimed at solubilizing poorly water-soluble drugs and to stabilize encapsulated therapeutic compounds to increase their delivery through cell membranes and biological barriers. For instance, Nifedipine and Cyclosporine are two examples of poorly water-soluble drugs. However, preparation of both compounds in aqueous solutions containing the fungal hydrophobin, SC3, results in an (i) increase of their solubility, (ii) improvement of their bioavailability, and consequently (iii) the long-lasting delivery of cyclosporine [191]. Some biomedical applications (e.g., diagnostics (i.e., imaging), gene therapy and drug delivery) require materials at the nanoscale range (i.e., nanomaterials) that possess high stability [192]. To generate self-emulsifying delivery systems, at the nanoscale range, surfactants have been used in formulations with good emulsifying properties controlling factors such as size, shape and stability of generated nanomaterial structures thus avoiding aggregation and allowing facilitation of a uniformed morphology [193,194,195]. In addition, surfactants such as (i) n-alkyl amine N-oxides (either on their own or in combination with lecithin), (ii) sugar surfactants (e.g., alkyl glucosides and sucrose), and (iii) polyglycerol fatty acid esters were shown to be safe and biodegradable [195]. Moreover, glycolipids are the biosurfactants mostly used in the synthesis and stabilization of nanoparticles (NPs). In addition, other biosurfactants reported to be acting as stabilizers and protecting agents, for silver NPs, are the rhamnolipids [196]. Finally, silver NPs synthesized from a laboratory biosurfactant (derived from *Pseudomonas aeruginosa* and produced from agro-industrial waste) have been reported to be “promising alternatives” to the ones using commercial rhamnolipids [197]. On the other hand, metallic (mainly gold and silver) NPs are heavily used in the biomedical field with emphasis in applications related to drug delivery, photodynamic and photodermal therapy and X-ray imaging and sensing (gold NPs), as well as in antimicrobial functions (silver NPs) [198,199]. To these ends, surfactin (produced by several strains of *Bacillus subtilis*) has been involved in the biological synthesis of gold and silver NPs [200,201] while sophorolipids have been reported as effective capping and reducing agents, in the synthesis of gold and silver NPs, without any observed cytotoxicity and/or genotoxicity when tested in *Fusarium oxysporum*, *Pseudomonas stutzeri* AG259, *Klebsiella penumoniae*, *Escherichia coli*, *Enterobacter cloacae*. However, these NPs were generated at a much lower rate than using purified surface-active agents in the reaction [202].

Biosurfactants can also be employed as plasticizers improving flexibility in solid dosage formulations, as lubricant, wetting agents and dispersants. The anti-adherent properties of some biosurfactants make them a reliable alternative for capsule and tablet formulations. The use of sucrose fatty acids as surfactants in tablet manufacturing has resulted in better flowability and disintegration than by adding magnesium stearate, a chemical surfactant normally used for tableting [203,204]. Furthermore, biosurfactants have been also characterized for transferring genes to cells and tissues in the context of medical applications [205]. For instance, mannosylerythritol lipid (MEL)-based cationic liposomes offer a promising way for gene delivery. This is a biosurfactant produced by the yeast strain *Candida antarctica* T-34 and has been shown to promote the interface of the liposome/nucleic acid transfection complex with a (-vely) charged cell membrane thus improving the overall gene transfection efficiency [206,207]. It has also been reported that cationic liposomes complexed to lipid helper cholesterol (L-dioleoylphosphatidylethanolamine (DOPE) together with the biosurfactant b-sitosterol b-D-glucoside) resulted in an improved efficiency of transferring the luciferase marker gene even in the presence of serum (which normally interferes with complex formation) [208].

Biosurfactant’s antimicrobial activity is also relevant to industrial and medical applications although currently have a limited use. Some biosurfactants (e.g., those obtained from *Pediococcus acidilactici* and *Lactobacillus plantarum* strains) have been shown to exert anti-adhesive and anti-microbial properties especially against *Staphylococcus aureus* CMCC26003 biofilm-related infections [209]. For instance, Daptomycin (a branched cyclic lipopeptide isolated from *Streptomyces roseosporus*) has reached a commercial use under the name of Cubicin^®^. This biosurfactant exerts bactericidal activity against all clinically relevant Gram (+ve) bacteria like vancomycin-resistant *enterococci* (VRE), methicillin-resistant *Staphylococcus aureus* (MRSA), intermediately susceptible *Staphylococcus aureus* (GISA), coagulase-negative *staphylococci* (CNS) and penicillin-resistant *Streptococcus pneumoniae* (PRSP). In addition, it can be helpful in treating endocarditis and bacteraemia caused by *S. aureus* as well as infections in the skin epidermis and the underlying soft tissues [210]. Finally, although marine bacteria are an unlimited source of biosurfactants they are difficult to grow under standard microbiological conditions which further limits the development of biodiscovery research. Despite that, several health-related properties have been shown for biosurfactants obtained from microorganisms growing under such conditions including anti-microbial, anti-adhesive and anti-biofilm activities against various human Gram (+ve) and Gram (−ve) bacteria as well as the yeast *Candida albicans*. In this context, several glycoproteins (derived from *Brevibacterium casei* MSA19, *Serratia marcescens*, *Spreptomyces* sp. B3, *Streptomyces* sp. MAB36, *Aspergillus ustus* MSF3) and lipopeptides (isolated from *Bacillus circulans* DMS-2, *Bacillus licheniformis* NIOT-AMKV06, *Brevibacterium aureum* MSA13, *Nocardiopsis alba* MSA10) were shown to act as antimicrobial agents [96]. However, in most of the cases, the antimicrobial activity of these glycopeptide and lipopeptide fractions were only partially characterized. Even more so, the biosurfactant composition that makes *Nocardiopsis dassonvillei* MAD08 to be effective against several organisms is still unknown since its antimicrobial activity was studied with the crude extracts rather than the purified molecule(s). On the other hand, the lipopeptide fraction produced by *Bacillus circulans* is the only one described to be effective against multidrug resistance (MDR) clinical isolates (*E. coli*, *K. pneumoniae* and *S. aureus*), thereby making this lipopeptide an alternative to existing drugs in treating infections caused by these pathogens [96].

Other biological properties reported for marine bacteria are related to tissue remodeling. For example, the polysaccharide HE800 EPS (produced by the bacteria *Vibrio diabolicus*) was found to restore injured bone in rats and to promote repair and healing of a connective tissue, in vitro. Furthermore, a branched acidic heteropolysaccharide EPS GY785 (isolated from *Alteromonas infernus*) induced the proliferation of chondrocytes grown on a hydrogel containing the biosurfactant [211].

## 4. Conclusions

In the last few years, there has been a tremendous interest in exploring the opportunity for producing microbial and/or marine-derived surface active agents (SAAs; e.g., biosurfactants), a class of molecules with the capacity to adsorb to fluid and solid/water interphases. In general, biosurfactants offer several advantages over their chemical counterparts, such as low toxicity, biodegradability, increased environmental compatibility, higher specificity and good stability. Moreover, marine surfactants have been shown to exert a wide array of biological properties including bacteriostatic and bactericidal (e.g., destabilizing the cell wall or outer membranes of the pathogens), anti-oxidant (e.g., scavenging of free radicals), anti-viral (e.g., functioning against viral attachment and entry), anti-inflammatory (e.g., reduction of pro-inflammatory mediators), anti-cancer (e.g., mediation of pro-apoptotic activities and cell cycle growth arrest) and anti-aging (e.g., protection of dermal elastic fiber network, etc.), all of which are essential in promoting human health. Consequently, marine-derived biosurfactants have been considered an integral part in a wide array of industrial applications towards end-user formulations in food, cosmetics, pharmaceutical/biomedical and other sectors, thereby offering a tremendous opportunity for more sustainable and biodegradable alternatives. In the context of blue bioeconomy, this translates into financial growth while, at the same time, meets consumers’ demand for ingredients of natural origin and contributes to increased environmental awareness.

## Figures and Tables

**Table 1:** Analytical approaches for evaluating the physicochemical characteristics of surfactants.

Techniques for Physicochemical Characterization	Physicochemical Characteristics Analyzed	Strengths	Limitations
Thin Layer Chromatography (TLC)	Qualitative analysis as well as polarity information of the molecules	Low cost and fast procedure	Soluble components of the mixtures could be detected
Mass Spectroscopy (MS)	Determination of MW	Accuracy and precision	Expensive equipment
	Structure elucidation	Accuracy, high sensitivity to detection and fast procedure	Lack of complete databases for identification purposes
Size Exclusion Chromatography (SEC)	Determination of MW and mixture separation	Enables separation and isolation of SAAs. Provides information about MW distribution	Expensive equipment
SEC-MALS SEC-Multiple Angle Light Scattering (MALS)	Determination of molecular radius and oligomerization state of high MW surfactants	Relatively accurate determination of absolute MW	Expensive equipment
Infrared Spectroscopy (IR) Attenuated Total Reflection—Fourier Transform Infrared (ATR-FTIR)	Provides structural information of surfactants	Fast and inexpensive process	Complicated sample preparation
		Minimal sample preparation	Interference and strong absorbance of H_2_O
Nuclear Magnetic Resonance (NMR)	Determination of size (indirect analysis), structure composition, purity and conformational change(s)	Non-invasive method and minimal sample preparation	Time consuming process. Large amount of sample is required

**Table 2:** Marine bacteria reported to produce bio-emulsifiers of interest to food manufacturers.

Organism	Emulsifier Structure	Properties	Reference
*Alteromonas* sp. Strain 1644	Anionic hetero-polysaccharide (glucose, galactose, mannose, rhamnose, glucuronic acid)	Thickening Gelation	[158]
*Halomonas* strain S30	Anionic hetero-polysaccharide (glucose, galactose, mannose, glucuronic acid)	Emulsification Thickening	[159]
*Alteromonas macleodii*	Anionic hetero-polysaccharide (glucose, galactose, glucuronic acid, galacturonic acid and pyruvate and acetate substituents)	Thickening	[160]
*Hahella chejuensis*	Heteropolysaccharide (galactose, glucose, xylose, ribose)	Emulsification Thickening	[161]
*Bacillus* sp. I-450	Anionic hetero-polysaccharide (galactose, fructose, glucose, raffinose, uronic acid, amino-sugars)	Thickening Gelation	[162]
*Vibrio harveyi* VB23	Heteropolysaccharide (galactose, glucose rhamnose, fucose, ribose, arabinose, xylose and mannose, uronic acids) and protein component	Emulsification	[163]
*Enterobacter cloacae*	Heteropolysacchride (fucose, galactose, glucose, glucuronic acid)	Emulsification	[164]
*Vibrio furnissii* VB0S3	Heteropolysaccharide (galactose, glucose, rhamnose, fucose, ribose, arabinose, xylose, mannose, uronic acids) and protein component	Emulsification	[165]
*Antarctobacter* sp. *TG22*	Anionic hetero-polysaccharide (rhamnose, fucose, galactose, galactosamine, glucose, glucosamine, mannose, muramic acid, galacturonic acid, glucuronic acid)	Emulsification	[166]
*Halomonas* sp, TG39 and TG67	Two anionic hetero-polysaccharides (rhamnose, fucose, galactose, galactosamine, glucose, glucosamine, mannose, xylose, muramic acid, galacturonic acid, glucuronic acid)	Emulsification	[167]
*Halomonas eurihalina* V2-7	Anionic hetero-polysaccharide. Protein and uronic acids	Emulsification Thickening	[168]
*Rhodococcus erythropolis* PR4	Anionic lipo-polysaccharide (galactose, glucose, mannose, glucuronic acid, pyruvic acid, esterified stearic, palmitic acids)	Emulsification	[169]
*Pseudoalteromonas* sp. TG12	Glycoprotein (rhamnose, fucose, galactose, galactosamine, glucose, glucosamine, mannose, xylose, muramic acid, galacturonic acid, glucuronic acid)	Emulsification	[170]
*Idiomarina fontislapidosi* F32, *I.ramblicola* R22	Anionic hetero-polysaccharide (glucose, mannose, galactose)	Emulsification	[171]
*Alteromonas hispanica* F23T	Anionic hetero-polysaccharide (glucose, mannose, xylose)	Emulsification	[170]
*Pseudoalteromonas ruthenica* SBT 033	Heteropolysaccharide (rhamnose, fructose, ribose, arabinose, xylose, mannose, galactose, glucose) containing uronic acid	Thickening	[172]
*Halomonas* sp. TG39	Anionic hetero-polysaccharide	Emulsification	[173]
*Flexibacter* sp. TG382	Glycoprotein	Emulsification Thickening	[174]
*Halomonas xianhensis* SUR308	Hetero-polysaccharide (glucose, galactose, mannose)	Thickening Heat stable	[175]
*Acinetobacter* sp.	Glyco-lipo-protein	Emulsification Surfactancy	[176]
*Acinetobacter bouvetii* UAM25	Exopolysaccharide	Emulsification	[177]
*Chromohalobacter canadensis* 28	Hetropolysaccharide (glucosamine, glucose, rhamnose, xylose), and protein (polyglutamate) complex	Emulsification Foaming Thickening Gelation	[178]
*Pseudomonas fluorescens*	Heteropolysaccharide (galactose, glucose, fructose, mannose, rhamnose) and protein	Emulsification	[179]
*Rhodobacter johrii* CDR-SL 7Cii	Heteropolysaccharide (glucose, glucuronic acid, rhamnose, galactose)	Emulsification Heat stable	[180]
